# Prognostic Discrimination of Alternative Lymph Node Classification Systems for Patients with Radically Resected Non-Metastatic Colorectal Cancer: A Cohort Study from a Single Tertiary Referral Center

**DOI:** 10.3390/cancers13153898

**Published:** 2021-08-02

**Authors:** Dimitrios Prassas, Pablo Emilio Verde, Carlo Pavljak, Alexander Rehders, Sarah Krieg, Tom Luedde, Wolfram Trudo Knoefel, Andreas Krieg

**Affiliations:** 1 Department of Surgery (A), Heinrich-Heine-University and University Hospital Duesseldorf, Moorenstr. 5, 40225 Duesseldorf, Germany; dimitrios.prassas@med.uni-duesseldorf.de (D.P.); carlo.pavljak@hhu.de (C.P.); rehders@med.uni-duesseldorf.de (A.R.); 2 Coordination Centre for Clinical Trials, Heinrich-Heine-University and University Hospital Duesseldorf, Moorenstr. 5, 40225 Duesseldorf, Germany; pablo.verde@med.uni-duesseldorf.de; 3 Clinic for Gastroenterology, Hepatology and Infectious Diseases, Heinrich-Heine-University and University Hospital Duesseldorf, Moorenstr. 5, 40225 Duesseldorf, Germany; sarah.krieg@med.uni-duesseldorf.de (S.K.); tom.luedde@med.uni-duesseldorf.de (T.L.)

**Keywords:** LODDS, LNR, lymph node classification, colorectal cancer

## Abstract

**Simple Summary:**

We compared the predictive and prognostic performance of different lymph node classification systems regarding overall survival in patients with colorectal cancer (CRC). Distinct lymph node ratio (LNR) and Log odds of positive lymph nodes (LODDS) classifications demonstrated prognostic superiority over the N category only in patients with Stage III CRC.

**Abstract:**

Background: Lymph node ratio (LNR) and the Log odds of positive lymph nodes (LODDS) have been proposed as a new prognostic indicator in surgical oncology. Various studies have shown a superior discriminating power of LODDS over LNR and lymph node category (N) in diverse cancer entities, when examined as a continuous variable. However, for each of the classification systems various cut-off values have been defined, with the question of the most appropriate for patients with CRC still remaining open. The present study aimed to compare the predictive impact of different lymph node classification systems and to define the best cut-off values regarding accurate evaluation of overall survival in patients with resectable, non-metastatic colorectal cancer (CRC). Methods: CRC patients who underwent surgical resection from 1996 to 2018 were extracted from our medical data base. Cox proportional hazards regression models and C-statistics were performed to assess the discriminative power of 25 LNR and 26 LODDS classifications. Regression models were adjusted for age, sex, extent of the tumor, differentiation, tumor size and localization. Results: Our study group consisted of 654 consecutive patients with non-metastatic CRC. C-statistic revealed 2 LNR and 5 LODDS classifications that demonstrated superior prognostic performance in patients with UICC III CRC, compared to the N category. No clear advantage of one classification over another could be demonstrated in any other patient subgroup. Conclusions: Distinct LNR and LODDS classifications demonstrate a prognostic superiority over the N category only in patients with Stage III radically resected CRC.

## 1. Introduction

Colorectal cancer (CRC) is one of the most common malignancies worldwide with an estimated number of approximately 148.000 new cases in the United States in 2020 [1]. Lymph node (LN) status is a significant prognostic factor, directly associated with disease free survival (DFS), as well as overall survival (OS) [2]. Its importance with regard to therapeutic decision making is deemed paramount, as LN metastasis constitutes an indication for perioperative treatment regimens, for most solid tumors. The most widely accepted standardized LN-staging system among clinicians is incorporated into the Tumor Node Metastasis (TNM) system maintained by the American Joint Committee on Cancer (AJCC) and the Union for International Cancer Control (UICC) [3,4]. In this system, cases with no metastatic LNs are classified as N0, cases with 1–3 metastatic LNs are classified as N1 and cases with more than 3 positive LNs are classified as N2. Moreover, N1 category is subdivided in N1a (1 metastatic LN), N1b (2–3 metastatic LNs) and N1c (no regional lymph nodes are positive but there are tumor deposits in the subserosa, mesentery or nonperitonealized pericolic or perirectal/mesorectal tissues), whereas N2 category is subdivided in N2a (4–6 metastatic LNs) and N2b (7 or more metastatic LNs). The minimum number of examined LNs (NELN) needed for an adequate staging, as recommended by the AJCC and UICC, should not be less than 12 in order to minimize the possibility of stage migration [5]. However, existing data that derive from population-based analysis suggest that cases with sufficient NELN can be as low as 37% of the study population [6]. The strong association between NELN and N category constitutes an inherent weakness of the TNM system and has necessitated the development of novel nodal staging systems that allow a better prognostic stratification. In this context, the metastatic LN Ratio (LNR: the number of positive LNs divided by the NELN) has been suggested during the last decade and has been evaluated in several studies, demonstrating superior independent prognostic value in CRC [7]. However, a major drawback of LNR becomes evident in node-negative disease, as it fails to deliver any more meaningful prognostic evaluation compared to TNM. An additional limitation of LNR is that cases in which all harvested LNs are positive are staged in the same class, regardless of the total number of harvested lymph nodes. It is clinically evident that LNR does not fully encompass the information contained in positive LNs and NELN. Log odds of positive lymph nodes (LODDS) is the natural logarithm of the ratio of the positive LNs and the negative LNs which has been reported to diminish the risk of stage migration in various types of solid malignancies [8,9,10]. Essentially, LODDS uses a mathematical approach to LN staging that is not influenced by the extent of lymphadenectomy thus representing the probability of a harvested LN to be metastatic. Existing data provide evidence on the suitability of LODDS as predictor for OS in CRC and other cancers. Moreover, it has been demonstrated that LODDS is an independent prognostic factor for survival in CRC patients [11]. However, the existing literature is characterized by notable diversity regarding cut-off points used to categorize the studied population into different subgroups. To date, these distinct cut-off points have not been compared and validated in independent sets of CRC patients. In the present study, we sought to shed light on this issue and determine the prognostically most appropriate set of, already proposed, cut-off points for different LN classification systems in patients with CRC that were treated at our department

## 2. Materials and Methods

### 2.1. Study Cohort

In the present study we retrospectively analyzed patient charts, histopathological findings and surgical reports collected from the prospectively maintained computer-based patient records database of the University Hospital Duesseldorf. Between November 1996 and August 2018, a total number of 996 adult patients with diagnosed primary CRC underwent surgery with curative intent at our department. Patients with the following criteria were excluded: metastatic disease (*n* = 148), incomplete histopathological information (*n* = 77), positive resection margins (*n* = 15), death within 30 days after surgery (*n* = 20), emergency surgery (*n* = 27), lost to follow up (*n* = 48) and polyposis syndromes and inflammatory bowel disease (*n* = 7) (Figure S1). All operations were performed via laparotomy. A high-tie of the central vessels was performed with a subsequent complete mesocolic excision. Regarding rectal resections, a total mesorectal excision was conducted. Circumferential resection margins could not be retrospectively retrieved for all cases of rectal cancer. OS was defined as time between date of surgery and death from any cause. All patients remained under outpatient follow-up of their oncological outcome where they were clinically examined by surgeons. The study was carried out in accordance with the principles of good clinical practice and the Declaration of Helsinki. Since this was a retrospective study, it was no longer possible to obtain a declaration of consent for data collection at a later date. For most of the patients, consent was no longer possible or involved a disproportionate amount of effort. In addition, all data analyzed were collected as part of routine diagnosis and treatment. The data were anonymized at the source and there was no evidence that the patients refused to use their data. An institutional review board (IRB)-approval of the Medical Faculty, Heinrich Heine University Duesseldorf was retrieved (IRB-No: 2019-428-ProspDEuA).

### 2.2. Tumor Staging and LN Classifications

Tumor stage was defined according to the TNM classification of malignant tumors 8th edition [4]. In cases where pathological tumor staging had already been conducted according to older TNM editions, it was appropriately converted to the 8th edition. The 8th edition system classifies LN involvement as N0 (no regional metastases), as N1 when 1-3 regional LN are positive and N2 when positive LNs are 4 or more [4]. LNR was calculated as the number of positive LNs divided by the NELN. LODDS was calculated by the empirical logistic formula: log[(number of positive LNs + 0.5)/(NELN − number of positive LNs + 0.5)]. LNR and LODDS were analyzed as both continuous and categorical variables. When used as categorical variables, different cut-off values were employed to subclassify the LN staging systems. For the LNR staging we used cut-off values from 25 different studies [2,10,11,12,13,14,15,16,17,18,19,20,21,22,23,24,25,26,27,28,29,30,31,32,33] whereas for the LODDS system, we used cut-off values as proposed by 26 different studies [2,10,12,14,15,16,18,19,20,21,23,24,27,28,32,33,34,35,36,37,38,39,40,41,42,43]. We included LNR and LODDS classifications from studies that were published until 31 December 2019. In addition, we defined a LODDS classification according to the percentile segments for LODDS in the study group. Accordingly, for our LODDS classification cut-off values were designated according to 25% and 75% percentiles (LODDS1: <25%, LODDS2: 25–50%, LODDS3: >50–75%, LODDS4: >75%).

### 2.3. Statistical Analysis

Scatter plots were designed to investigate the relationship between the number of metastatic lymph nodes, LNR and LODDS. The accuracy of various LN classifications was analyzed as a continuous variable by measuring the area under the receiver operating characteristics (ROC) curve (AUC) using SPSS statistics for Windows (IBM SPSS Statistics for Windows, Version 25.0. Armonk, NY, USA: IBM Corp.). Kaplan–Meier curves were generated and compared by the log-rank (Mantel-Cox) test using GraphPad Prism for Windows (Version 8.0.2, GraphPad Software, San Diego, CA, USA).

The relationship between distinct cut-off values of different LN classifications systems and OS was analyzed using a multivariate Cox proportional regression model calculating the hazard ratio (HR) and 95% confidence intervals (CI). Therefore, we fitted a base model including the following covariates: age at the time of surgery, sex, T-category (T1 + 2, T3 + 4), degree of histologic differentiation (well/moderately differentiated, G1 + G2; poorly differentiated/undifferentiated G3 + G4), tumor size and localization (rectum, right/transverse/left/sigmoid colon).

Using this model, we further evaluated for each LN classification model discrimination using the C-statistics. We compared two C-statistics by using the same data set and by calculating the jackknife variance estimates of the difference between two C-statistics. The comparison is made by interpreting the 95% CI of the difference of the C-statistics. The Delta C parameter was calculated in order to quantify the differences of C-statistic between the N category and any other classification. The false discovery rate (FDR) method was used to adjust the *p*-values resulting in the comparison between the N category and any other classification.

In addition, we performed the above-mentioned analysis in subgroups defined by the presence of lymph node metastases (UICC I/II versus UICC III), history of a neoadjuvant therapy (yes versus no), tumor localization (rectum versus right/transverse/left/sigmoid colon) and number of resected LNs (≥12 versus <12). For the sample size determination, we assumed a constant hazard rate (HR) of 1.5 between the low and higher N-grading classes during the complete follow-up period of 150 months. When the total sample size is 420 with a total number of events required of 210, a 0.05 level two-sided log-rank test for equality of survival curves will have 90% power to detect a difference between the two groups. The total number of patients of *n* = 654 in our cohort with 252 observed events are enough to detect a statistically significant difference between N-grading classification groups.

For risk factors with missing data, we used a simple imputation method using medians for continuous variables and the most often frequency for categorical outcomes. Statistical analysis was performed using the statistical software R version 3.6.3 [44]. We used reporting tools based on the standards of replicable research using the R package “knitr” [45]. The analysis based on the proportional hazard Cox’s regression and the estimation of the C-statistics was performed with the R package “survival” [46].

## 3. Results

A total number of 654 consecutive patients with non-metastatic CRC could be included in our study. Baseline clinical and histopathological characteristics of the study population are summarized in Table 1.

**Figure 1:**
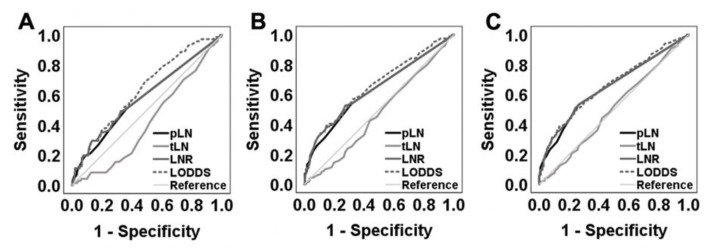
ROC analysis of various LN classification systems. ROC curves were generated for LNR, LODDS, pLN (positive lymph nodes), tLN (total lymph nodes) as categorical variables to predict (**A**) 1-year OS, (**B**) 3-year OS and (**C**) 5-year OS.

There were 403 (61.6%) patients without LN metastases. The median (range) of NELN and positive LNs in the whole cohort was 17 (2–68) and 0 (0–56), respectively. The study population consisted of 239 (36.5%) rectal cancer patients and 415 (63.5%) cases of colon cancer. First, we evaluated various LN classification systems as categorical variables by conducting a ROC analysis for 1-year, 3-year and 5-year OS (Figure 1A–C).

**Figure 2:**
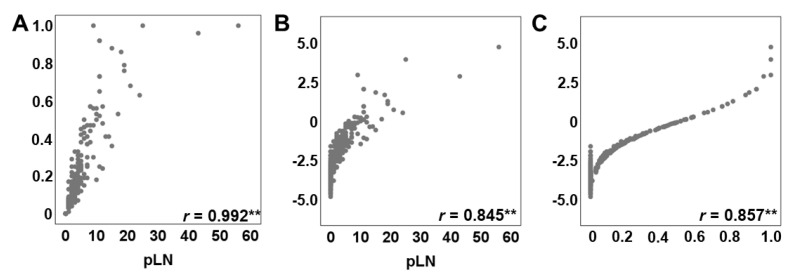
Relationship between positive lymph nodes (pLN), LNR and LODDS. Scatter plots presenting the distribution of (**A**) LNR versus pLN, (**B**) LODDS versus pLN and (**C**) LNR versus LODDS. ** *p* < 0.001.

Accordingly, LODDS was the only LN classification exhibiting the highest AUC values with a *p* < 0.001 during all three follow up phases (Table S1). In addition, we displayed LN parameters in a scatter plot to verify the relationship between the numbers of metastatic lymph nodes (positive LN, pLN), LNR and LODDS (Figure 2A–C).

Scatter plots demonstrated that both, LNR and LODDS, increased with the number of pLN (r_s_ = 0.992, r_s_ = 0.845). Moreover, LODDS also increased with LNR values (r_s_ = 0.857). However, when LNR was 0 or 1, LODDS remained heterogeneous implying that LODDS discriminates more precisely among patients without lymph node metastasis and patients in which the number of pLN is equal to the NELN. Of note, although we observed a tendency towards a more favorable prognosis in patients with a NELN ≥ 12, this difference became not statistically significant (Figure S2). We then performed Kaplan–Meier survival analysis for the AJCC 8th edition N-staging as well as the distinct LNR and LODDS classification systems demonstrating a ubiquitous statistically significant association with OS (Figures S3–S5).

To further analyze the highest discriminative power of the different LN staging systems in predicting prognosis we performed Cox proportional hazards regression and evaluated model discrimination for each LN parameter using the overall C index. Therefore, we first examined the prognostic value of the selected covariates in our base model using Cox regression analysis. Accordingly, age at the time of surgery, tumor size, grade of tumor differentiation and tumor localization were significantly associated with OS (Table 2).

Using this base model, we performed for each LN classification system Cox regression analysis and evaluated model discrimination using the C-statistics in our entire cohort of CRC patients. Cox regression analysis revealed that advanced N categories as well as higher LNR or LODDS categories, even independently of the different cut-off values, were significantly associated with a poor prognosis (Tables S2–S4). However, C-statistics demonstrated comparable results for all classification systems showing no superiority of any LNR or LODDS classification when compared with the AJCC 8th edition N category (Figure S6).

Postoperative chemotherapy has been shown to significantly improve survival of stage III CRC patients [47]. Given the side effects of the currently administered chemotherapeutics, a shorter chemotherapy for selected patients characterized by a lower risk of recurrence would be desirable. In this context, Grothey and colleagues demonstrated that for CRC patients with a low risk situation (T1, T2, or T3 and N1) a therapy of 3 months with CAPOX (capecitabine and oxaliplatin) was not inferior to 6 months [48]. In contrast, for high risk patients (T4, N2 or both) a therapy of 6 months was superior when compared with a regimen of 3 months. In addition, the JFMC37-0801 study revealed superior recurrence free and overall survival in stage III B, III C and IV CRC patients for 12 months of capecitabine [49]. Accordingly, among UICC III CRC patients there exist subgroups with a lower risk that is reflected by a better prognosis. This prompted us to investigate whether LNR and LODDS classification systems may further prognostically discriminate risk groups within the subgroup of UICC III cancer patients. Interestingly, C-statistics revealed that 2 LNR [2,21] and 5 LODDS [2,20,23,24,35] classifications exhibited a superior discrimination in OS when compared with the N category (Table 3).

**Figure 3:**
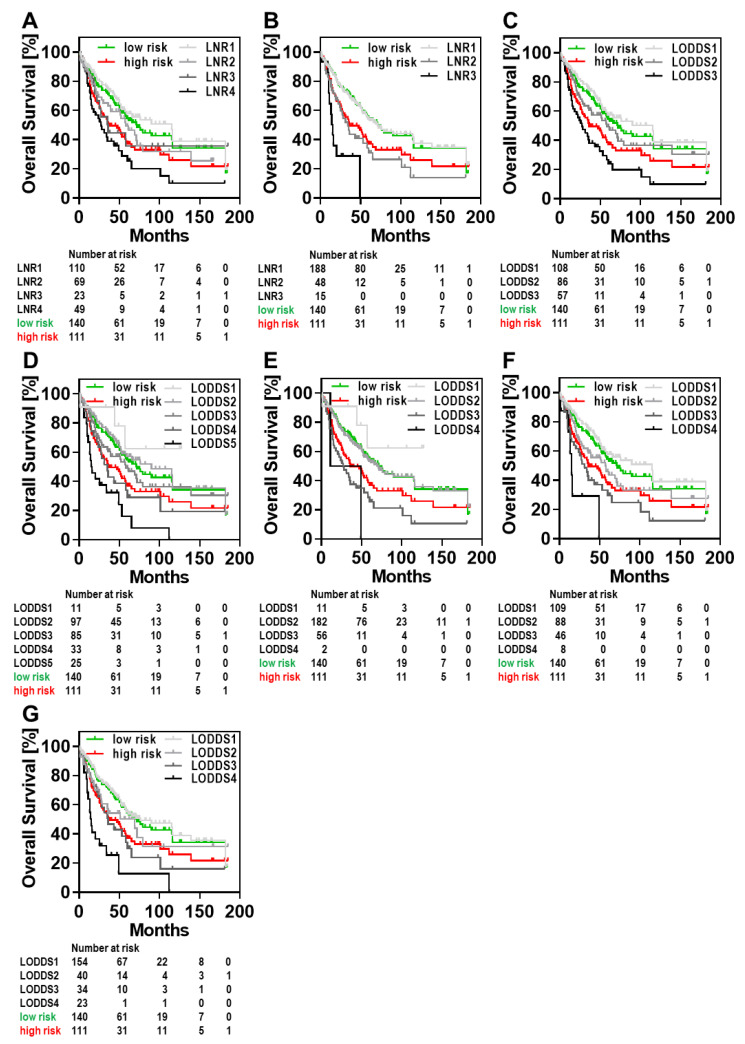
Kaplan–Meier survival curves for OS in UICC III CRC patients (*n* = 251) depending on the LNR classification system proposed by (**A**) Lee et al. [21], (**B**) Fortea-Sanchis et al. [2] or the LODDS classification system suggested by (**C**) Fortea-Sanchis et al. [2], (**D**) He et al. [35], (**E**) Calero et al. [20], (**F**) Bagante et al. [23] and (**G**) Jian-Hui et al. [24]. Red and green OS curves indicate high risk (T4, N2 or both) and low risk (T1, T2, or T3 and N1) patients, respectively.

Consistent with these observations, survival curves for low and high risk patients either perfectly matched with the survival curves of LNR groups 1 and 2 defined by Fortea-Sanchis [2] or demonstrated at least a parallel course with the survival curves of certain LNR or LODDS groups of the remaining 6 LN classifications [2,20,21,23,24,35] in UICC III CRC patients (Figure 3). This observation suggests that these 7 LN-classification systems might serve as a useful tool in the decision making of the duration of an adjuvant chemotherapy.

Of note, in other subgroups defined by the tumor localization, history of neoadjuvant radio-chemotherapy, NELN LNR and LODDS classifications failed to demonstrate a prognostic superiority (data not shown).

## 4. Discussion

The role of LN metastasis in the systemic dissemination of CRC is crucial. LN status is regarded as one of the major prognostic parameters for assessing the course of the disease after CRC resection. Along with the TNM classification maintained by the AJCC/UICC, based on the positive LN category (N) further LN classification systems have been developed. This is a result of the limitations of the N category, as it is solely based on the number of positive LNs, regardless of the radicality of locoregional lymphadenectomy. The LNR system was introduced as an alternative to N category as it takes into account not only the positive LNs but also the total number of harvested LNs. However, an inherent limitation of the above-mentioned classification is the heterogeneity of patients in cases where all resected LNs are positive or when all resected LNs are negative. LODDS has been therefore introduced as a LN classification system that resolves this issue and is defined as the logarithm of the ratio between the probability of being a metastatic LN and the probability of being a negative harvested LN, when a LN is retrieved. The prognostic value of LNR and LODDS has already been evaluated in patients with CRC and other types of cancer, also in patients who underwent emergency surgery for complicated CRC [50]. Both, LNR and LODDS are continuous biological variables. Nevertheless, such variables are of little use or, in the worst case, cannot be applied in clinical practice. As a result, a plethora of categorical cut-off values for various LN staging systems have been proposed. The remarkable heterogeneity of existing cut-off values is a consequence of clinical and/or methodological diversity among the existing studies. In our study, the prognostic impact of 25 LNR and 27 LODDS classifications was investigated in patients following curative-intent resection of CRC. After confirming the predictive value of LNR and LODDS in our patient cohort as a continuous variable, we further sought to compare the various LN classifications as a categorical variable using C-statistics, based on already-published cut-off values. However, in our study cohort, none of the proposed sets of cut-off values were able to demonstrate superiority over the N category. Exclusively in the subgroup of UICC III CRC patients, 2 LNR [2,21] and 5 LODDS [2,20,23,24,35] classifications demonstrated a predictive superiority when compared with the N category. Of note, stage III CRC patients constitute a distinctive subgroup of cases that require the administration of adjuvant chemotherapy. However, the usual regimen of 6-month treatment is associated with cumulative neurotoxicity and, as a result, in quality of life deterioration. Hitherto, the issue of balance between choice of regimen, therapy duration and risk of toxicity has been addressed in various trials [48,51]. Ivenson et al. [51] conducted the largest single randomized study on adjuvant treatment of CRC and clearly demonstrated the non-inferiority of 3-month oxaliplatin-based regimen versus the standard 6-month duration. On the other hand, in the study of Grothey et al. [48], a large prospective pooled analysis of six randomized Phase III trials, two different risk groups of UICC III CRC patients were identified in which a 3-month CAPOX-regimen was noninferior to 6 months of chemotherapy regarding disease free survival. Furthermore, a study from Japan randomly assigned patients with radically resected UICC III stage CRC to oral adjuvant chemotherapy for 6 or 12 months, demonstrating an improved OS in the 12-month treatment group for advanced UICC III B, C and UICC IV stages [49].

Accordingly, our results provide valuable data for the further subclassification of stage III CRC patients in distinctive risk groups. That is of utmost importance as we verified which novel LN classification systems could be implemented in the tailored decision-making process of selecting the most appropriate duration of adjuvant therapy regimen for the suitable subgroup of patients.

To our knowledge, our study is the first attempt to directly compare previously proposed LN classification systems. We now provide novel data, which generate the basis for future research and point the direction to the evaluation of specific LN classification systems that appear to have clinical and therapeutic relevance in patients with UICC III CRC. However, there is a number of inherent limitations to all cohort studies of this type. The patients represented a selected cohort that were radically operated in a highly specialized setting and are consequently not representative of all patients diagnosed with CRC. Cohort size was modest and disease free survival was not recorded. Moreover, administration of neoadjuvant radiochemotherapy in patients with rectal cancer has not been consistent over the observed period and thus, the results regarding this subgroup should be interpreted with caution. Additionally, the administration of adjuvant therapy could not be fully evaluated within this retrospective study. Data regarding exact chemotherapeutic drugs administered, their dosage, frequency and duration are incomplete. At this point it must also be stated that all surgeries were performed via laparotomy, which was our institutional standard during the study period, and thus explains the lack of laparoscopic and/or robotic approaches in our study. Howbeit, the amount of total harvested lymph nodes has not been found to differ significantly between these three different surgical approaches in the existing literature [52,53,54,55,56].

The strengths of the study, nevertheless, included robust follow-up data with a reasonable duration of follow-up. Patients were recruited from a consecutive series diagnosed with CRC, from a single geographical region, all treated by the same group of specialists, using a standardized staging algorithm and operative techniques.

## 5. Conclusions

In conclusion, LNR cut-off values as proposed by Lee et al. [21] and Fortea-Sanchis et al. [2], as well as LODDS classifications as proposed by Fortea-Sanchis et al. [2], He et al. [35], Calero et al. [20], Bagante et al. [23] and Jian-Hui et al. [24] demonstrate a clear prognostic superiority over the N category in the subgroup of patients with UICC III CRC. Therefore, we believe that future examination of LN classification systems with cut-off values other than the above mentioned should be abandoned in patients with UICC III CRC and that focus should be turned on the further verification of our findings, in the context of larger-scale clinical trials.

## Figures and Tables

**Table 1:** Patient characteristics.

Variable	Overall	UICC I	UICC II	UICC III
Number of subjects	654	171	232	251
Age				
Median (range)	70 (20–98)	70 (37–89)	69 (25–98)	71 (20–96)
Gender				
Male	364 (55.7)	102 (59.6)	129 (55.6)	133 (53.0)
Female	290 (44.3)	69 (40.4)	103 (44.4)	118 (47.0)
Surgery type				
Right hemicolectomy	178 (27.2)	41 (24)	63 (27.2)	74 (29.5)
Extended right hemicolectomy	32 (4.9)	4 (2.3)	20 (8.6)	8 (3.2)
Transverse colectomy	12(1.8)	2 (1.2)	6 (2.6)	4 (1.6)
Extended left hemicolectomy	13 (2.0)	4 (2.3)	7 (3.0)	2 (0.8)
Left hemicolectomy	40 (6.1)	11 (6.4)	11 (4.7)	18 (7.2)
Sigmoid colectomy	104 (15.9)	24 (14.0)	42 (18.1)	38 (15.1)
Anterior resection	216 (33)	72 (42.1)	59 (25.4)	85 (33.9)
Abdominoperineal resection	24 (3.7)	7 (4.1)	11 (4.7)	6 (2.4)
others	35 (5.4)	6 (3.5)	13 (5.6)	16 (6.4)
Tumor location				
Caecum	59 (9.0)	14 (8.2)	19 (8.2)	26 (10.4)
Ascending colon	127 (19.4)	28 (16.4)	22 (22.0)	48 (19.1)
Transverse colon	64 (9.8)	9 (5.3)	35 (15.1)	20 (8.0)
Descending colon	37 (5.7)	14 (8.2)	7 (3.0)	16 (6.4)
Sigmoid colon	112 (17.1)	24 (14.0)	47 (20.3)	41 (16.3)
Rectum	239 (36.5)	80 (46.8)	69 (29.7)	90 (35.9)
Synchronous tumors	16 (2.4)	2 (1.2)	4 (1.7)	10 (4.0)
T stage				
T1	51 (7.8)	45 (26.3)		6 (2.4)
T2	155 (23.7)	126 (73.7)		29 (11.6)
T3	383 (58.6)		207 (89.2)	176 (70.1)
T4	65 (9.9)		25 (10.8)	40 (15.9)
N stage				
N0	403 (61.6)	171 (100)	232 (100)	
N1	163 (24.9)			163 (64.9)
N2	88 (13.5)			88 (35.1)
No. of examined LN, median (range)	17 (2–68)	15 (3–53)	17 (2–62)	19 (6–68)
No. of positive LN, median (range)	0 (0–56)	0	0	2 (0–56) *
Tumor differentiation				
G1	17 (2.6)	7 (4.1)	6 (2.6)	4 (1.6)
G2	524 (80.1)	146 (85.4)	190 (81.9)	188 (74.9)
G3	101 (15.4)	14 (8.2)	31 (13.4)	56 (22.3)
G4	1 (0.2)	1 (0.6)	0 (0)	0 (0)
Unknown	11 (1.7)	3 (1.8)	5 (2.2)	3 (1.2)
Neoadjuvant treatment				
No	590 (90.2)	149 (87.1)	205 (88.4)	236 (94)
Yes	64 (9.8)	22 (12.9)	27 (11.6)	15 (6.0)

* One patient included with N1c (no regional lymph nodes were positive but there were pericolorectal tumor deposits).

**Table 2:** Cox regression analysis of the variables considered for the multi-variable adjusted base model.

Risk Factor	HR (95% CI)	*p*
Age		
Age < 70	1.00 (reference)	<0.001
Age ≥ 70	3.32 (2.53–4.36)	
Gender		
Female	1.00 (reference)	0.108
Male	1.23 (0.95–1.59)	
Tumor size		
<median	1.00 (reference)	0.044
≥median	1.32 (1.01–1.73)	
Grading		
G1 + G2	1.00 (reference)	0.001
G3 + G4	1.69 (1.23–2.30)	
*T stage*		
T1 + T2	1.00 (reference)	1.017
T3 + T4	1.43	
Tumor		
localization		
Colon	1.00 (reference)	0.005
Rectum	1.46 (1.12–1.89)	

**Table 3:** Summary of the LN classification systems that demonstrate better prognostic discrimination than the N category in UICC III CRC patients.

LN Classification	HR (95% CI)	C-Index (95% CI)	Delta C	P_c_
LNR Lee et al. [21]		0.6955 (0.6668–0.7242)	0.0166	0.037
≥0; ≤0.1	1.000 (Reference)			
>0.1; ≤0.2	1.321 (0.836–2.087)			
>0.2; ≤0.3	1.947 (1.006–3.767)			
>0.3	2.133 (1.349–3.373)			
LNR Fortea-Sanchis et al. [2]		0.6995 (0.6708–0.7282)	0.0207	0.031
0; 0.24	1.000 (Reference)			
0.25; 0.60	1.811 (1.191–2.754)			
>0.60	3.514 (1.785–6.915)			
LODDS Fortea-Sanchis et al. [2]		0.7020 (0.6732–0.7308)	0.0231	0.027
<−2	1.000 (Reference)			
≥−2; ≤−1	1.299 (0.840–2.008)			
>−1	2.387 (1.538–3.707)			
LODDS He et al. [35]		0.7023 (0.6735–0.7311)	0.0235	0.036
<−3	1.000 (Reference)			
≥−3; <−2	1.397 (0.425–4.600)			
≥−2; <−1	1.794 (0.541–5.953)			
≥−1; <0	2.363 (0.685–8.157)			
≥0	5.457 (1.563–19.054)			
LODDS Calero et al. [20]		0.6998 (0.6710–0.7286)	0.0210	0.036
≤−3	1.000 (Reference)			
>−3; ≤−1	1.552 (0.481–5.011)			
>−1; ≤3	3.195 (0.962–10.607)			
>3	3.324 (0.526–21.012)			
LODDS Bagante et al. [23]		0.6977 (0.6710–0.7290)	0.0188	0.038
<−2	1.000 (Reference)			
≥−2; ≤−0.9	1.413 (0.921–2.168)			
>0.9; ≤1.5	2.162 (1.354–3.453)			
>1.5	3.396 (1.390–8.297)			
LODDS Jian-Hui et al. [24]		0.7080 (0.6790–0.7370)	0.0180	0.027
≤−1.5	1.000 (Reference)			
>−1.5; ≤−1	1.233 (0.740–2.054)			
>−1; ≤0	1.619 (0.992–2.641)			
>0	4.324 (2.441–7.658)			

## Data Availability

The data presented in this study are available on request from the corresponding author. The data are not publicly available due to ethical issues.

## Supplementary Materials

The following are available online at https://www.mdpi.com/article/10.3390/cancers13153898/s1, Figure S1: Case selection for outcome analysis, Figure S2: Kaplan–Meier curves for OS depending on the number of resected lymph nodes (NLN), Figure S3: Kaplan–Meier survival curves for OS depending on the N category (AJCC/UICC 8th edition), Figure S4: Kaplan–Meier survival curves for OS depending on the LNR classification system reported by (A) Arslan et al. [11], (B) Malleo et al. [13], (C) Sun et al. [10], (D) Xu et al. [12], (E) Zhou et al. [8], (F) Riediger et al. [15], (G) Lee et al. [21], (H) Negi et al. [22], (I) Bagante et al. [23], (J) Jian-Hui et al. [24], (K) Smith et al. [25], (L) Calero et al. [20], (M) Fang et al. [16], (N) Wang et al. [17], (O) Conci et al. [18], (P) Huang et al. [19], (Q) Liu et al. [26], (R) Chang et al. [27], (S) Song et al. [28], (T) Wang et al. [33], (U) La Torre et al. [29], (V) Agnes et al. [30], (W) Rosenberg et al. [31], (X) Cao et al. [32], (Y) Fortea-Sanchis et al. [2]., Figure S5: Kaplan–Meier survival curves for OS depending on the LODDS classification system reported by (A) He et al. [35], (B) Fortea-Sanchis et al. [2], (C) Ramacciato et al. [36], (D) Toth et al. [37], (E) Xu et al. [12], (F) Yang et al. [34], (G) Zhou et al. [14], (H) Sun et al. [10], (I) Xu et al. [38], (J) Riediger et al. [15], (K) Fang et al. [16], (L) Conci et al. [18], (M) Huang et al. [19], (N) Calero et al. [20], (O) Cao et al. [32], (P) Lee et al. [21], (Q) Cao et al. [39], (R) Amini et al. [40], (S) Bagante et al. [23], (T) Jian-Hui et al. [24], (U) Wu et al. [41], (V) Wang et al. [42], (W) Chang et al. [27], (X) Song et al. [28], (Y) Persiani et al. [43], (Z) Wang et al. [33], or (A1) according to the percentiles, Figure S6: Forrest plot of the C-statistics with 95% confidence intervals depending on the LN classification system in the entire study group of CRC patients, Table S1: ROC analysis of various LN classification systems for 1- year, 3-year and 5-year OS, Table S2: OS depending on the N category defined by the AJCC/UICC, Table S3: OS depending on the respective LNR classification. Each LNR subgroup is defined by an LNR range as indicated, Table S4: OS depending on the respective LODDS classification. Each LODDS subgroup is defined by a LODDS range as indicated. * The LODDS3 subgroup proposed by Ramacciato et al. [36] containing LODDS values between 0.012 and 0.026 was omitted as none of our included patients exhibited LODDS value within this range.
