# Supplementary material for: Prognostic Discrimination of Alternative Lymph Node Classification Systems for Patients with Radically Resected Non-Metastatic Colorectal Cancer: A Cohort Study from a Single Tertiary Referral Center
Source: Cancers (Basel). 2021 Aug 2;13(15):3898. doi: 10.3390/cancers13153898 (PMC8345552; doi:10.3390/cancers13153898)
Supplement: Supplementary file 1 [file cancers-13-03898-s001.zip › Table S1.pdf]

Table S1. ROC analysis of various LN classification systems for 1- year, 3-year and 5-year OS.

**Overall Survival (OS)**	**LN evaluation**	**ROC analysis**		
	**Type**	**AUC**	**95% CI**	***p*-value**
1-year OS	LODDS	0.634	0.567 – 0.701	< 0.001
	LNR	0.591	0.516 – 0.666	0.013
	tLN	0.421	0.356 – 0.486	0.030
	pLN	0.583	0.509 – 0.657	0.023
3-year OS	LODDS	0.658	0.606 – 0.710	< 0.001
	LNR	0.640	0.586 – 0.695	< 0.001
	tLN	0.472	0.420 – 0.525	0.311
	pLN	0.632	0.578 – 0.686	< 0.001
5-year OS	LODDS	0.660	0.609 – 0.711	< 0.001
	LNR	0.653	0.602 – 0.704	< 0.001
	tLN	0.502	0.449 – 0.556	0.933
	pLN	0.648	0.597 – 0.699	< 0.001
