# Supplementary material for: Prognostic Discrimination of Alternative Lymph Node Classification Systems for Patients with Radically Resected Non-Metastatic Colorectal Cancer: A Cohort Study from a Single Tertiary Referral Center
Source: Cancers (Basel). 2021 Aug 2;13(15):3898. doi: 10.3390/cancers13153898 (PMC8345552; doi:10.3390/cancers13153898)
Supplement: Supplementary file 1 [file cancers-13-03898-s001.zip › Table S2.pdf]

Table S2. OS depending on the N category defined by the AJCC/UICC

**N Classification AJCC/UICC 8th edition**	**Positive Lymph Nodes**	**HR**	**95%CI**
0	0	1	(Reference)
1	1-3	1.634	1.206-2.214
2	≥4	2.675	1.899-3.768
