# Supplementary material for: Prognostic Discrimination of Alternative Lymph Node Classification Systems for Patients with Radically Resected Non-Metastatic Colorectal Cancer: A Cohort Study from a Single Tertiary Referral Center
Source: Cancers (Basel). 2021 Aug 2;13(15):3898. doi: 10.3390/cancers13153898 (PMC8345552; doi:10.3390/cancers13153898)
Supplement: Supplementary file 1 [file cancers-13-03898-s001.zip › Table S3.pdf]

Table S3. OS depending on the respective LNR classification. Each LNR subgroup is defined by a LNR range as indicated.

LNR Classification	Subgroup	LNR Range	HR	95% CI
Agnes et al.[30]	1	0	1	(Reference)
	2	0.01; 0.10	1.438	1.013-2.041
	3	0.11; 0.25	1.979	1.361-2.876
	4	0.26; 0.40	3.832	2.171-6.762
	5	> 0.40	3.153	2.008-4.951
Arslan et al.[11]	1	≤ 0.05	1	(Reference)
	2	>0.05-0.2	1.523	1.117-2.076
	3	> 0.20	2.957	2.080-4.204
Bagante et al.[23]	1	0	1	(Reference)
	2	0.01; 0.25	1.671	1.250-2.235
	3	0.26; 0.5	2.415	1.506-3.872
	4	> 0.5	6.794	3.949-11.688
Calero et al.[20]	1	0	1	(Reference)
	2	0.01; 0.25	1.656	1.238-2.215
	3	0.26; 0.75	3.076	2.049-4.617
	4	>0.75	5.526	2.584-11.817
Cao et al.[32]	1	0; 0.24	1	(Reference)
	2	0.25; 0.28	1.658	1.243-2.213
	3	>0.28	3.368	2.290-4.953
Chang et al.[27]	1	≤ 0.08	1	(Reference)
	2	0.09; 0.17	1.479	0.984-2.224
	3	0.18; 0.33	2.961	1.909-4.592
	4	> 0.33	2.699	1.796-4.055
Conci et al.[18]	1	0	1	(Reference)
	2	>0; ≤0.25	1.642	1.228-2.195
	3	>0.25	3.340	2.289-4.873
Fang et al.[16]	1	< 0.1	1	(Reference)
	2	0.1; 0.33	2.034	1.464-2.827
	3	≥ 0.34	2.717	1.809-4.082
Fortea-Sanchis et al.[2]	1	0; 0.24	1	(Reference)
	2	0.25; 0.60	2.466	1.679-3.623
	3	> 0.60	4.635	2.424-8.860
Huang et al.[19]	1	< 0.25	1	(Reference)
	2	≥0.25; <0.5	2.078	1.330-3.251
	3	≥0.50; <0.75	4.648	2.546-8.486
	4	≥0.75	4.330	2.061-9.097
Jian-Hui et al.[24]	1	0	1	(Reference)
	2	0.01; ≤ 0.1	1.436	1.011-2.039
	3	> 0.1; ≤ 0.25	1.980	1.363-2.878
	4	> 0.25	3.374	2.311-4.926

La Torre et al.[29]	1	0	1	(Reference)
	2	0.010; 0.199	1.593	1.184-2.143
	3	0.200; 0.399	3.225	1.970-5.280
	4	> 0.399	3.251	2.083-5.072
Lee et al.[21]	1	≤ 0.1	1	(Reference)
	2	>0.1; ≤ 0.2	1.746	1.189-2.565
	3	>0.2; ≤ 0.3	2.665	1.459-4.868
	4	>0.3	2.893	1.970-4.250
Liu et al.[26]	1	0	1	(Reference)
	2	0.01; 0.10	1.437	1.012-2.040
	3	0.11; 0.40	2.264	1.613-3.178
	4	> 0.40	3.183	2.026-4.999
Malleo et al.[13]	1	0	1	(Reference)
	2	>0; ≤0.2	1.607	1.194-2.162
	3	>0.2; 0.4	3.082	1.903-4.991
	4	>0.4	3.115	7.985-4.890
Negi et al.[22]	1	0	1	(Reference)
	2	> 0; ≤ 0.5	1.782	1.354-2.345
	3	> 0.5	6.789	3.948-11.675
Riediger et al.[15]	1	< 0.1	1	(Reference)
	2	0.1; 0.199	1.742	1.180-2.574
	3	≥ 0.2	2.765	1.976-3.869
Rosenberg et al.[31]	1	0	1	(Reference)
	2	0.01; 0.17	1.543	1.137-2.094
	3	0.18; 0.41	3.026	1.982-4.618
	4	0.42; 0.69	2.851	1.673-4.858
	5	≥0.70	4.938	2.323-10.496
Smith et al.[25]	1	0	1	(Reference)
	2	>0; ≤ 1/15	1.545	1.044-2.286
	3	>1/15; ≤ 3/10	1.843	1.323-2.569
	4	>3/10; ≤ 7/10	2.908	1.881-4.496
	5	> 7/10	4.913	2.312-10.441
Song et al.[28]	1	0	1	(Reference)
	2	0.01; 0.11	1.482	1.051-2.088
	3	0.12; 0.36	2.330	1.635-3.320
	4	0.37; 0.66	2.453	1.500-4.013
	5	> 0.66	5.410	2.640-11.087
Sun et al.[10]	1	0	1	(Reference)
	2	0.01; 0.2	1.635	1.215-2.201
	3	0.21; 0.5	2.375	1.557-3.623
	4	> 0.5	6.814	3.960-11.726
Wang et al.[17]	1	0	1	(Reference)
	2	0.01; 0.30	1.728	1.298-2.301
	3	0.31; 0.60	2.658	1.685-4.193
	4	0.61; 1	5.913	3.039-11.507

Wang et al.[33]	1	< 0.07	1	(Reference)
	2	0.07; < 0,25	1.483	1.078-2.041
	3	0.25; < 0,50	2.327	1.471-3.681
	4	0.50; 1	5.103	3.103-8.392
Xu et al.[12]	1	0	1	(Reference)
	2	>0; ≤ 0.125	1.501	1.073-2.101
	3	> 0.125; ≤ 0.425	2.219	1.564-3.148
	4	> 0.425; ≤ 1	3.366	2.131-5.316
Zhou et al.[8]	1	0; ≤0.30	1	(Reference)
	2	>0.3; ≤0.7	2.309	1.526-3.496
	3	>0.7; ≤1	3.674	1.759-7.673
