# Supplementary material for: Prognostic Discrimination of Alternative Lymph Node Classification Systems for Patients with Radically Resected Non-Metastatic Colorectal Cancer: A Cohort Study from a Single Tertiary Referral Center
Source: Cancers (Basel). 2021 Aug 2;13(15):3898. doi: 10.3390/cancers13153898 (PMC8345552; doi:10.3390/cancers13153898)
Supplement: Supplementary file 1 [file cancers-13-03898-s001.zip › Table S4.pdf]

Table S4. OS depending on the respective LODDS classification. Each LODDS subgroup is defined by a LODDS range as indicated. *The LODDS3 subgroup proposed by Ramacciato et al.[36] containing LODDS values between 0.012 and 0.026 was omitted as none of our included patients exhibited LODDS value within this range.

**LODDS Classification**	**Subgroup**	**LODDS Range**	**HR**	**95%CI**
Percentiles	1	≤ -3.611	1	(Reference)
	2	> -3.611; ≤ -3.219	1.182	0.798-1.751
	3	> -3.219; ≤ -2.162	1.679	1.124-2.507
	4	> -2.162	2.693	1.861-3.898
Amini et al.[40]	1	≤ -3	1	(Reference)
	2	> -3; < 0	1.839	1.400-2.415
	3	≥ 0	5.812	3.502-9.645
Bagante et al.[23]	1	< -2	1	(Reference)
	2	- 1.99; -0.90	1.888	1.342-2.658
	3	- 0.89; 1.50	2.898	1.954-4.299
	4	> 1.5	4.605	1.973-10.745
Calero et al.[20]	1	≤ -3	1	(Reference)
	2	>-3; ≤-1	1.723	1.300-2.291
	3	>-1; ≤3	3.614	2.461-5.307
	4	>3	3.735	0.894-15.612
Cao et al.[32]	1	≤ -0.5	1	(Reference)
	2	> -0.5; ≤ 0	1.238	0.630-2.428
	3	> 0; ≤ 0.5	6.353	2.930-13.777
	4	> 0.5	4.350	2.280-8.297
Cao et al.[39]	1	≤ -2.6	1	(Reference)
	2	> -2.6; ≤ -1.6	1.529	1.110-2.107
	3	> -1.6; ≤ -0.5	2.379	1.625-3.483
	4	> -0.5	3.164	2.052-4.878
Chang et al.[27]	1	≤ -0.92	1	(Reference)
	2	-0.91; -0.62	6.530	3.032-14.061
	3	-0.61; -0.26	1.633	0.761-3.505
	4	> - 0.26	2.693	1.723-4.210
Conci et al.[18]	1	≤ -1.35	1	(Reference)
	2	> -1.35; ≤ -1	1.517	0.795-2.892
	3	> -1; ≤ -0.25	3.092	1.864-5.127
	4	> -0.25	2.783	1.778-4.359
Fang et al.[16]	1	< -0.82	1	(Reference)
	2	≥ -0.82; < -0.57	5.594	2.697-11.604
	3	≥ -0.57	2.418	1.624-3.599
Fortea-Sanchis et al.[2]	1	<-2	1	(Reference)

	2	≥ -2; ≤ -1	1.753	1.235-2.487
	3	> -1	3.211	2.240-4.605
He et al.[35]	1	< -3	1	(Reference)
	2	≥ -3; < -2	1.554	1.114-2.167
	3	≥ -2; < -1	2.046	1.413-2.963
	4	≥ -1 ; < 0	2.694	1.655-4.386
	5	≥ 0	5.896	3.550-9.792
Huang et al.[19]	1	< -1	1	(Reference)
	2	≥ -1.00; < 0	2.087	1.310-3.325
	3	≥ 0; < 1	4.640	2.542-8.470
	4	≥ 1,00; < 2	5.930	2.089-16.831
	5	≥ 2	3.389	1.225-9.377
Jian-Hui et al.[24]	1	≤ -1.5	1	(Reference)
	2	> -1.5; ≤ -1	1.739	1.082-2.796
	3	> -1; ≤ 0	2.237	1.429-3.501
	4	> 0	5.751	3.399-9.733
Lee et al.[21]	1	≤ -4	1	(Reference)
	2	> -4; ≤ -2.5	1.809	1.045-3.132
	3	> -2.5; ≤ -2	2.039	1.093-3.806
	4	> -2; ≤ -0.5	3.511	1.965-6.274
	5	> -0.5	4.952	2.612-9.388
Persiani et al.[43]	1	≤ -1.36	1	(Reference)
	2	-1.35; -0.53	2.620	1.691-4.060
	3	≥ -0.52	2.452	1.633-3.681
Ramacciato et al.[36]*	1	<-0.005	1	(Reference)
	2	≥ -0.005; <0.012	1.894	0.459-7.812
	4	≥ 0.026	4.955	2.955-8.309
Riediger et al.[15]	1	< -1	1	(Reference)
	2	-1; -0.500	3.588	2.023-6.365
	3	-0.499; -0.001	1.168	0.545-2.501
	4	≥0	4.513	2.778-7.334
	5	> -0.5	4.952	2.612-9.388
Song et al.[28]	1	≤ -2.51	1	(Reference)
	2	> -2.51; ≤ -1.68	1.395	0.983-1.979
	3	> -1.68; ≤ -0.51	2.240	1.559-3.221
	4	> -0.51; ≤ 0.73	2.678	1.637-4.383
	5	> 0.73	4.722	2.231-9.997
Sun et al.[10]	1	≤ -1.5	1	(Reference)
	2	-1.49; -1.000	1.754	1.091-2.820
	3	-0.999; -0.500	4.056	2.275-7.230
	4	-0.499; 0	1.352	0.687-2.659
	5	> 0	5.846	3.453-9.898
Toth et al.[37]	1	< -1.125	1	(Reference)
	2	-1.125; -0.251	2.539	1.586-4.065
	3	-0.250; 0.749	2.101	1.224-3.606
	4	≥ 0.750	3.800	1.818-7.945

Wang et al.[33]	1	< -2.2	1	(Reference)
	2	-2.2; < -1.1	1.660	1.199-2.299
	3	-1.1; < 0	2.207	1.410-3.454
	4	0; < 1.1	5.258	2.8596-9.668
	5	≥ 1.1	5.082	2.396-10.777
Wang et al.[42]	1	≤ -2.5	1	(Reference)
	2	> -2.5; ≤ -1	1.539	1.144-2.070
	3	> -1; ≤ 0.9	3.141	2.113-4.670
	4	> 0.9	4.608	2.178-9.749
Wu et al.[41]	1	≤ -1.46	1	(Reference)
	2	- 1.45; -1.17	2.114	1.185-3.772
	3	-1.16; - 0.73	3.819	2.136-6.827
	4	≥ -0.72	2.531	1.709-3.748
Xu et al.[12]	1	≤ -2.8	1	(Reference)
	2	> -2.8; ≤ -1.60	1.493	1.087-2.050
	3	> -1.60; ≤ -0.31	2.376	1.637-3.449
	4	> -0.31	3.361	2.129-5.307
Xu et al.[38]	1	< -1	1	(Reference)
	2	-1.000; -0.501	3.594	2.025-6.379
	3	-0.500; -0.001	1.169	0.546-2.503
	4	0; 0.499	4.358	2.204-8.617
	5	≥ 0.500	4.662	2.440-8.908
Yang et al.[34]	1	≤ -1.43	1	(Reference)
	2	> -1.43; ≤ -1.20	2.164	1.126-4.159
	3	> -1.20; ≤ -0.69	2.966	1.696-5.188
	4	> -0.69	2.515	1.699-3.724
Zhou et al.[14]	1	≤ -1	1	(Reference)
	2	-0.999; -0.500	3.737	2.104-6.637
	3	-0.499; 0.500	1.996	1.191-3.346
	4	> 0.500	4.638	2.428-8.862
