# Supplementary figures and images for: Prognostic Discrimination of Alternative Lymph Node Classification Systems for Patients with Radically Resected Non-Metastatic Colorectal Cancer: A Cohort Study from a Single Tertiary Referral Center
Source: Cancers (Basel). 2021 Aug 2;13(15):3898. doi: 10.3390/cancers13153898 (PMC8345552; doi:10.3390/cancers13153898)

## Slide 1
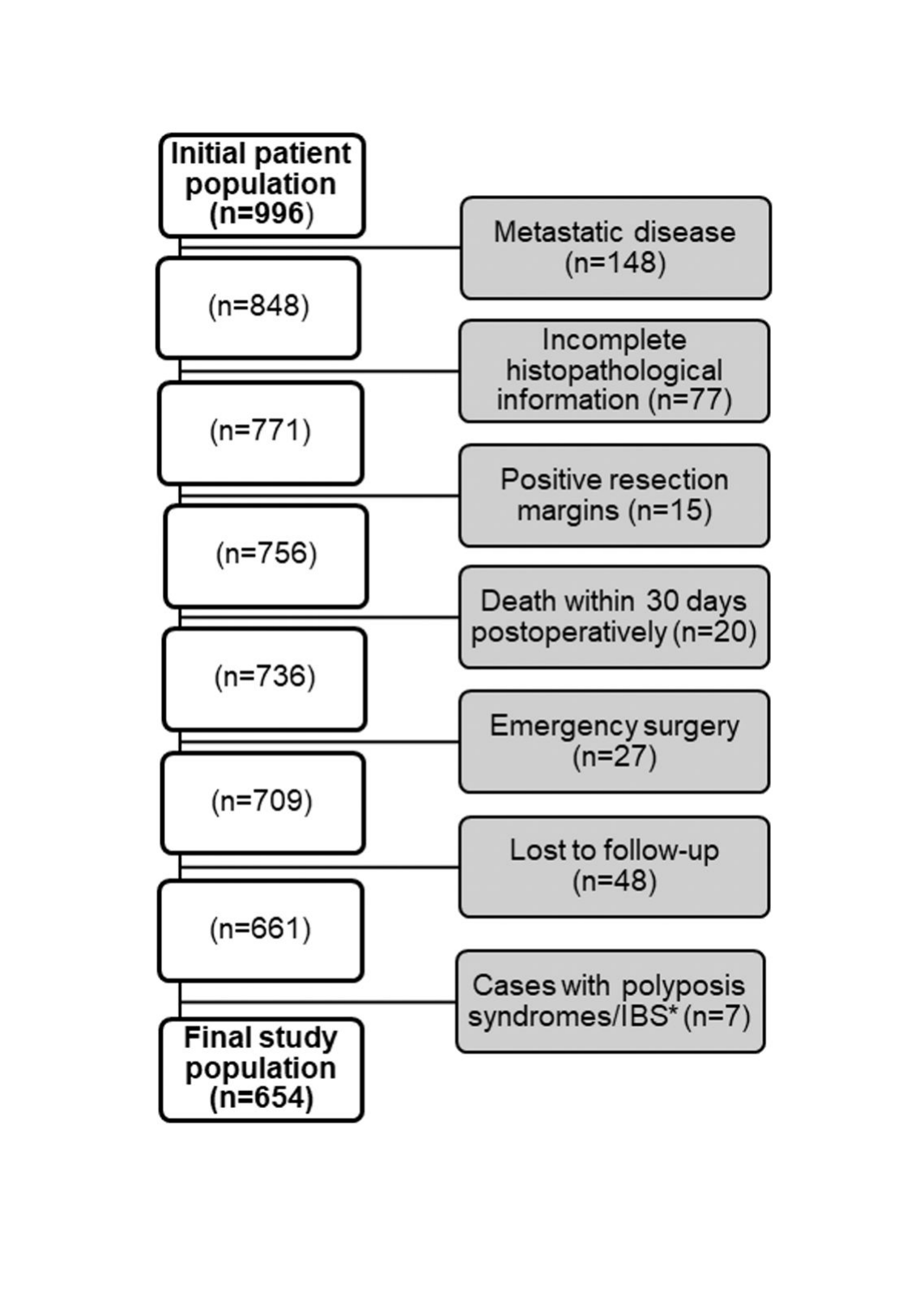

Supplement: Supplementary file 1 [file cancers-13-03898-s001.zip › Figure S1.pptx]

## Slide 1
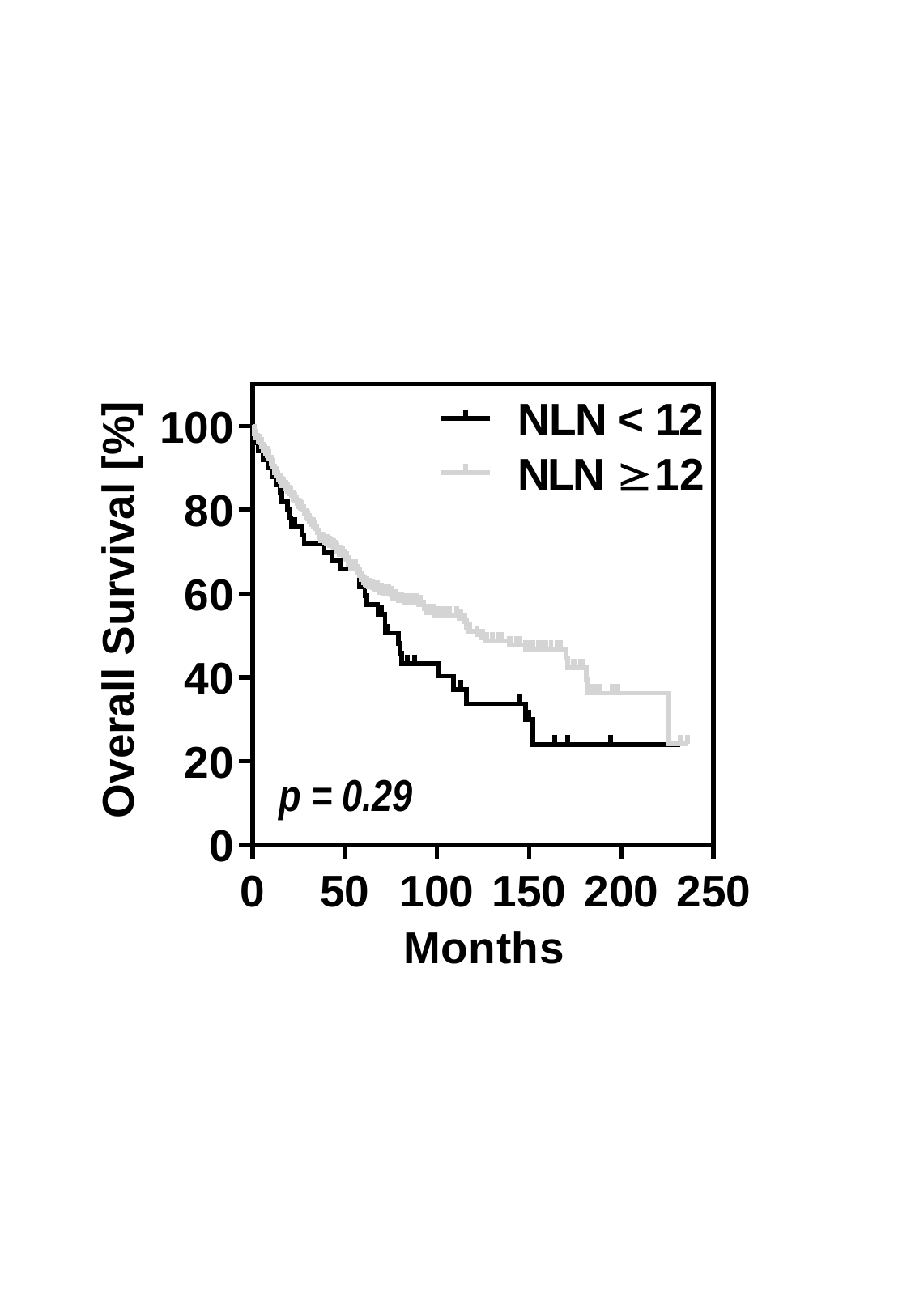

Supplement: Supplementary file 1 [file cancers-13-03898-s001.zip › Figure S2.pptx]

## Slide 1
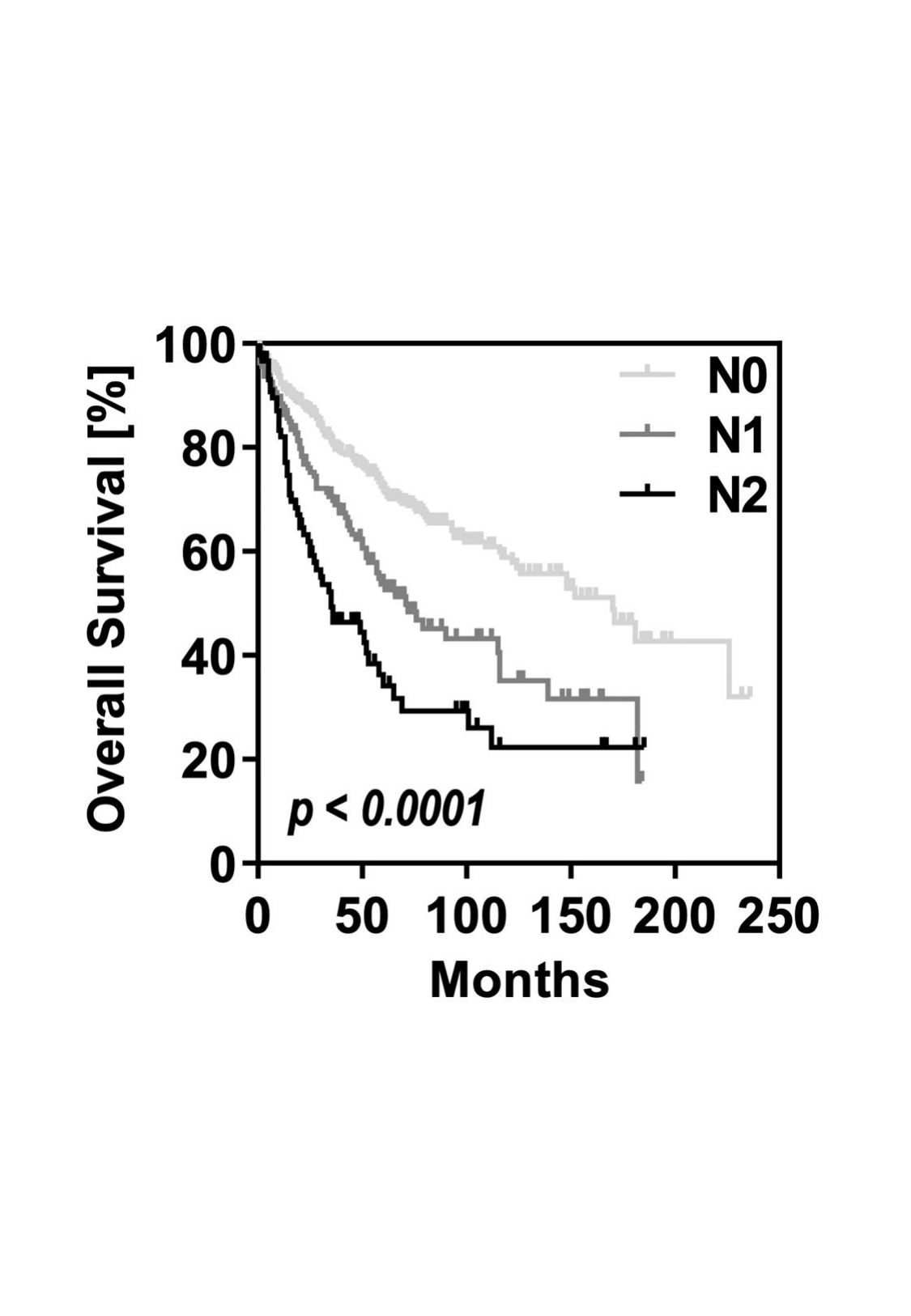

Supplement: Supplementary file 1 [file cancers-13-03898-s001.zip › Figure S3.pptx]

## Slide 1
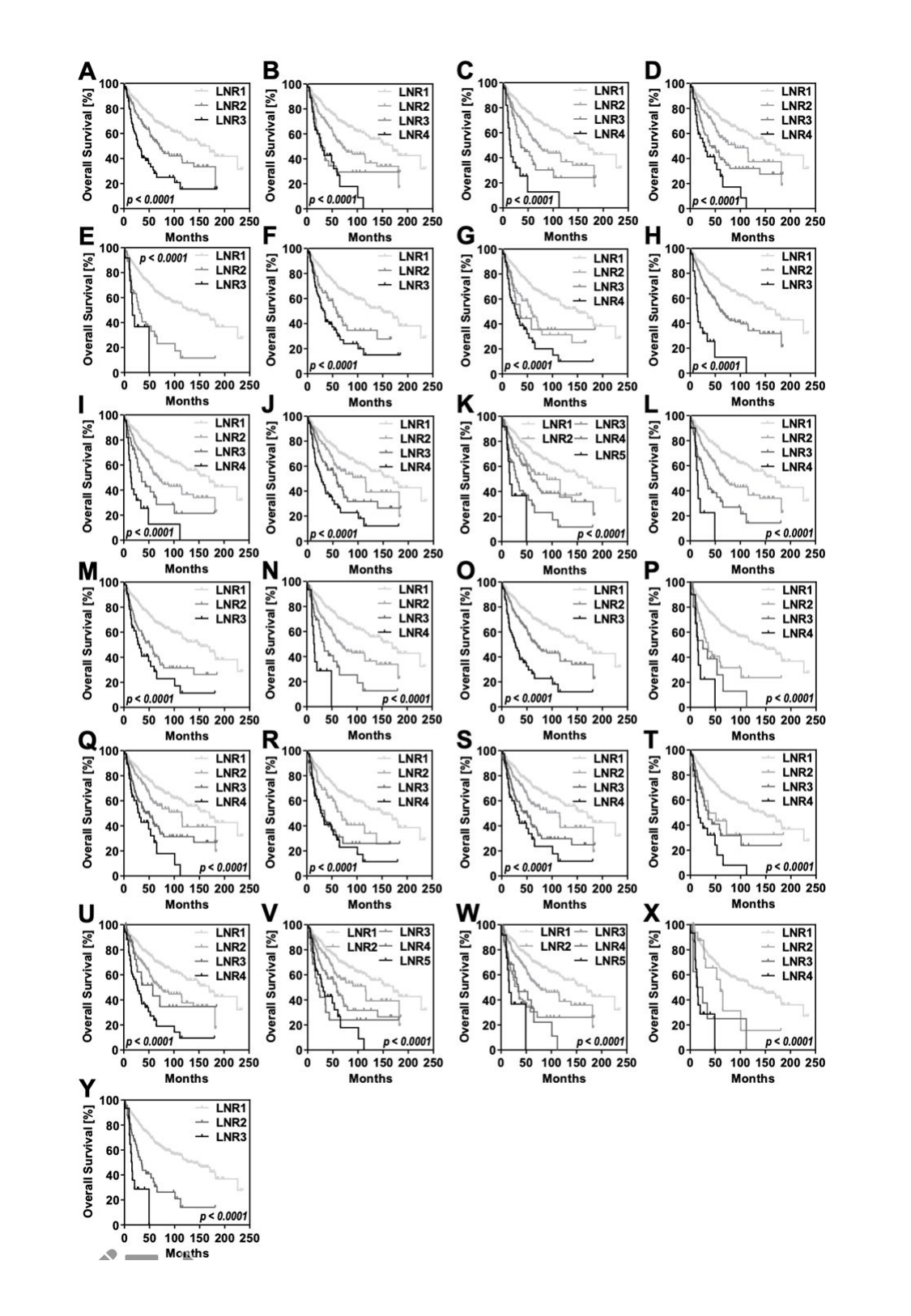

Supplement: Supplementary file 1 [file cancers-13-03898-s001.zip › Figure S4.pptx]

## Slide 1
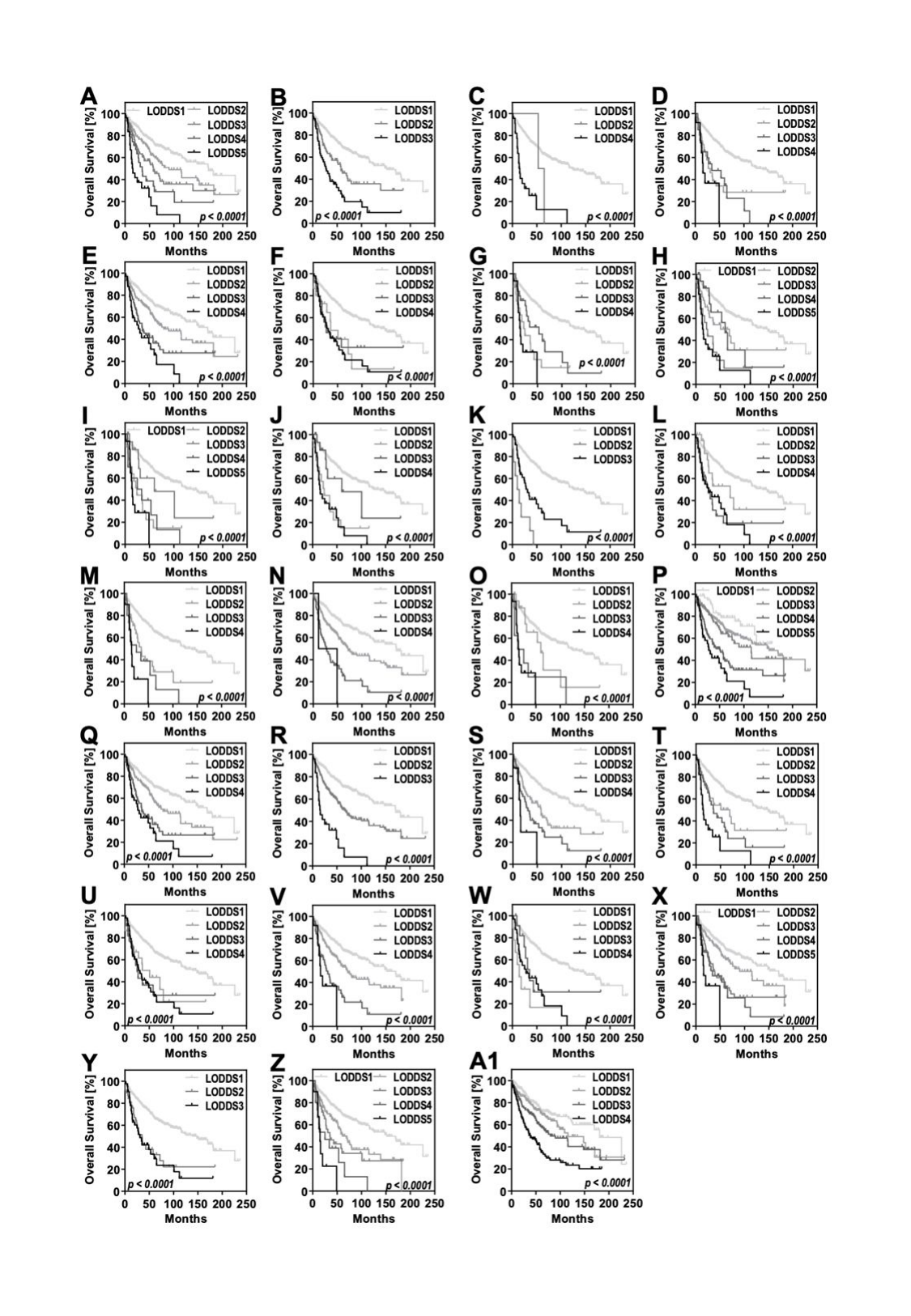

Supplement: Supplementary file 1 [file cancers-13-03898-s001.zip › Figure S5.pptx]

## Slide 1
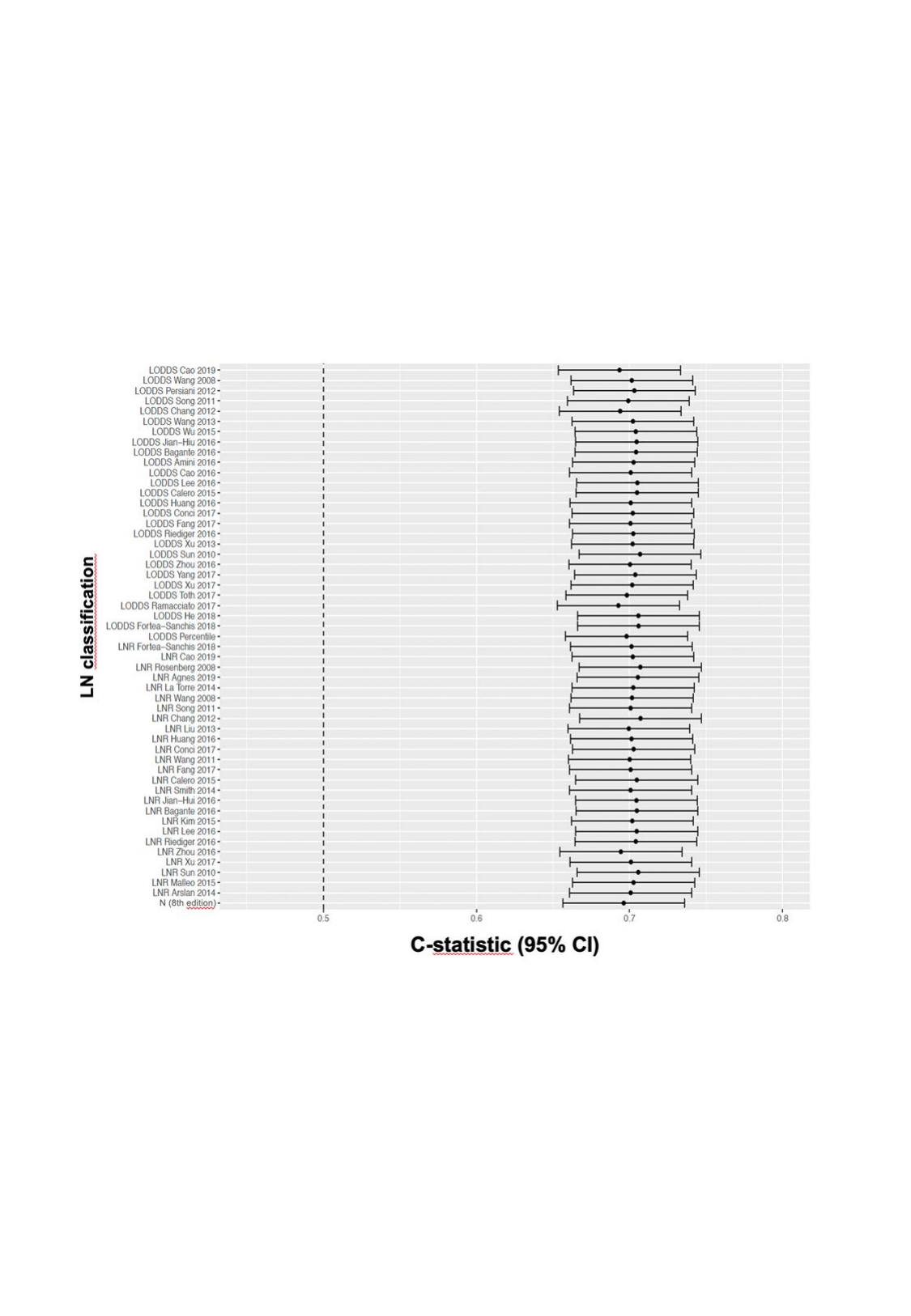

Supplement: Supplementary file 1 [file cancers-13-03898-s001.zip › Figure S6.pptx]
